# Assessing the Health Impact of Malaria Control Interventions in the MDG/Sustainable Development Goal Era: A New Generation of Impact Evaluations

**DOI:** 10.4269/ajtmh.17-0509

**Published:** 2017-09-27

**Authors:** Alexander K. Rowe

**Affiliations:** Malaria Branch, Division of Parasitic Diseases and Malaria, Centers for Disease Control and Prevention, Atlanta, Georgia

## INTRODUCTION

Malaria remains a major cause of preventable death. The World Health Organization estimated that malaria killed 429,000 people (uncertainty range: 235,000–639,000) in 2015, with most deaths occurring in sub-Saharan Africa and among children under 5 years old.^1^ Nearly two decades ago, the Roll Back Malaria (RBM) partnership was created to help combat this plague. The initial targets were to halve malaria mortality from 2000 to 2010 and again from 2010 to 2015.^2^ In addition, the Millennium Development Goals (MDGs) included a target of reducing malaria incidence by 2015. With global incidence falling by an estimated 37% compared with 2000, this target has been achieved.^3^ Current targets, which correspond to the era of the Sustainable Development Goals (SDGs), are to reduce malaria incidence and mortality by 90% from 2015 to 2030.^4^ More than US$ 20 billion has been dedicated to the fight over the past decade, with an estimated US$ 2.9 billion spent on malaria control and elimination in 2015.^1^

Ambitious RBM and MDGs, along with the substantial funding that these goals have attracted, made it important to conduct impact evaluations to show how much malaria control efforts had actually reduced malaria mortality. However, impact evaluations are challenging because measuring malaria mortality directly (i.e., counting all malaria deaths) is not feasible in most of sub-Saharan Africa. The earliest impact evaluations in Africa were sub-national malaria control and research projects, such as those conducted from the 1950s to 1970s in Kenya, Tanzania, and Nigeria.^5–7^ The relatively small scale of these projects allowed for intensive, high-quality, community-based monitoring of child mortality and entomological measures of malaria transmission, which are generally not feasible for national-level programs.

Around the time of the establishment of RBM, a number of reports considered practical methods for evaluating the impact of national-level malaria control programs,^8–13^ which led to one of the first impact evaluations in the MDG era, in Zanzibar.^14^ In 2007, RBM’s Monitoring and Evaluation Reference Group published a framework for evaluating the impact of malaria control efforts on mortality in sub-Saharan Africa.^15^ The framework informed additional impact evaluations.^16–18^

This supplement to the *American Journal of Tropical Medicine and Hygiene* includes nine contributions on the evaluation of the impact of malaria control interventions. The purposes of this supplement are to highlight the successes of malaria control efforts and present new methods for evaluating the impact of large-scale malaria control programs.

## SUPPLEMENT OVERVIEW

Yé et al. have updated RBM’s decade-old framework for evaluating the impact of malaria control efforts.^19^ This expanded framework includes new features, such as examining high-risk subpopulations most likely to demonstrate improvement from intervention scale-up, using a national platform framework, and analyzing complete birth histories from national household surveys to characterize the association between exposure to malaria control interventions and all-cause child mortality (ACCM).

The article by Hershey et al.^20^ is a companion piece to the new RBM framework.^19^ The authors have reflected on their experiences with planning and conducting impact evaluations in nearly a dozen countries, and they offer practical lessons and recommendations that should influence and benefit future impact evaluations, such as ensuring country ownership, engaging and coordinating stakeholders and partners, tailoring evaluations to country contexts, and using a standard methodology and informative dissemination products.

Thomson et al. explore ways to use data on rainfall and temperature (two potentially important confounders of the association between malaria intervention scale-up and reductions in malaria burden) in the evaluation of national malaria control programs in Africa.^21^ They use a new quality-controlled dataset on rainfall and temperature and other climate information to assess the likely effect of climate on impact evaluations in 10 countries.

Ashton et al. present methods for using data from routine health management information systems (HMIS) for impact evaluations, with a focus on improving internal validity, reducing bias of impact estimates, and the identification of quasi-experimental designs that are particularly well-suited for HMIS data, such as interrupted time series and dose-response analyses.^22^ This article should be the go-to reference for analysts using HMIS data.

Ng et al. used multiple data sources from Zambia to produce district-level estimates of intervention coverage, contextual factors, and ACCM, and they then fit a model that estimated mortality trends as interventions were scaled-up.^23^

Two articles examined malaria control efforts in Malawi. Florey et al. evaluated the impact of the national scale-up of insecticide-treated nets (ITNs) on ACCM with two complementary methods: a retrospective cohort analysis of individual children and a district-level ecologic analysis—both of which controlled for numerous confounders.^24^ Hershey et al. evaluated the combined effect of all major malaria control interventions on a broader array of impact indicators: malaria parasitemia, severe anemia, as well as ACCM.^25^

In Senegal^26^ and Rwanda,^27^ the evaluations take advantage of widely varying malaria transmission levels to show greater programmatic impact in areas with higher malaria transmission. Such comparisons strengthen the causal link between malaria intervention scale-up and reductions in malaria morbidity and mortality. This approach is especially important because these countries experienced increasing coverage of non-malaria interventions (e.g., vaccines, breastfeeding, and improved water sources) and general economic improvements that likely contributed to ACCM reductions. The Rwanda evaluation is also notable for the use of decomposition modeling, which estimated the contribution of various factors (e.g., bed net ownership) to reductions in ACCM.

These studies convey three important messages. First, they demonstrate an increased sophistication in the use of national cross-sectional household surveys and of multiple data sources. Taken together, the articles represent a conceptual and practical guide for planning and executing a new generation of impact evaluations. This guidance might be of value for evaluating the impact of programs for other health conditions in low-resource settings. Second, the studies illustrate the substantial time and effort required to conduct a successful evaluation, including the engagement of many experts to accommodate an array of analytic approaches. It is heartening to see how these evaluations can help build in-country capacity on programmatic monitoring and evaluation.^20^ Third, the studies demonstrate the tremendous impact of malaria interventions.

## CHALLENGES AND THE WAY FORWARD

Although this supplement includes some very fine work, the validity of future impact evaluations could be improved further, and to do so requires tackling several important methodologic challenges. First, in the context of large historical declines in ACCM in most countries, the counterfactual (what would have occurred in the absence of malaria control) needs to be better estimated. Second, programs and advocates would surely appreciate a clearer quantification of programmatic impact on malaria-related deaths, which would also require standard methods for calculating the uncertainty of impact estimates. Third, limitations exist for the high-quality household surveys that are used in all evaluations, such as recall bias and failure to collect the full range of data needed for a given analysis (e.g., data on ITNs discarded before the survey visit, retrospective ITN use by individual children, or immunization coverage among children who died).^24^ Fourth, better data are needed on the uptake and impact of case-management, which is a major pillar of all malaria control programs. Fifth, we need to find better ways of weaving practical impact evaluations into routine programs. Future impact evaluations should be anticipated, with needed data collected and analyzed prospectively, so the time to produce a formal impact evaluation is reduced. Continuous household and health facility surveys, for example, might allow for future impact evaluations to be done more quickly, updated regularly (e.g., every 1–2 years), and reflect the effect of scaling-up malaria case-management.^28^ Finally, despite improvements in many countries, the fact that routine HMIS data often still have poor (or unknown) validity and limited representativeness remains an important obstacle, especially for monitoring future progress toward SDGs. Weak HMIS data was a theme throughout the entire supplement. Timely, valid, and representative HMIS data are, of course, also useful for managing programs and essential for malaria elimination efforts. With improved HMIS data and optimal intervention coverage, and eventually the achievement of malaria elimination, we will finally know the precise proportion of malaria deaths prevented: 100%.
